# Supplementary material for: Danon Disease: Entire LAMP2 Gene Deletion with Unusual Clinical Presentation—Case Report and Review of the Literature
Source: Genes (Basel). 2023 Jul 27;14(8):1539. doi: 10.3390/genes14081539 (PMC10454823; doi:10.3390/genes14081539)
Supplement: Supplementary file 1 [file genes-14-01539-s001.zip › genes-2456230-supplementary/Supplementary FIGURE and LAMP2 MUTATIONS EXCEL FILES/Figure S1.pdf]

Supplementary Figure S1

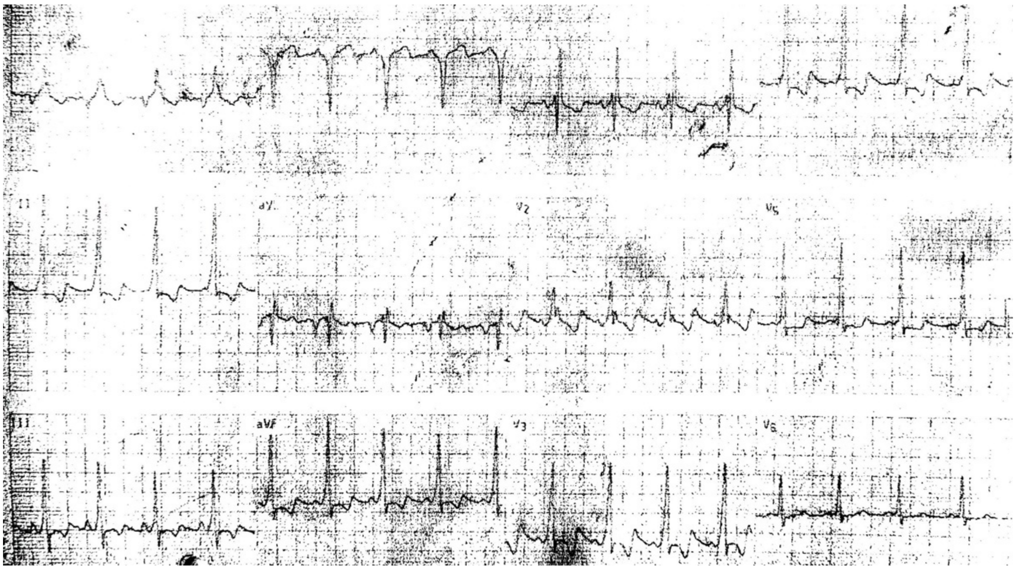

The Case's 1 ECG results at the age of 15 years old, showing the Wolf Parkinson white (WPW) pattern.
